# Impact of Aging on the 6-OHDA-Induced Rat Model of Parkinson’s Disease

**DOI:** 10.3390/ijms21103459

**Published:** 2020-05-14

**Authors:** Sandra Barata-Antunes, Fábio G. Teixeira, Bárbara Mendes-Pinheiro, Ana V. Domingues, Helena Vilaça-Faria, Ana Marote, Deolinda Silva, Rui A. Sousa, António J. Salgado

**Affiliations:** 1 Life and Health Sciences Research Institute (ICVS), School of Medicine, University of Minho, Campus de Gualtar, 4710-057 Braga, Portugal; id7405@alunos.uminho.pt (S.B.-A.); fabioteixeira@med.uminho.pt (F.G.T.); id7153@alunos.uminho.pt (B.M.-P.); id8807@alunos.uminho.pt (A.V.D.); helena.mv.faria@gmail.com (H.V.-F.); id5820@alunos.uminho.pt (A.M.); id7403@alunos.uminho.pt (D.S.); 2 ICVS/3B’s—PT Government Associate Laboratory, 4710-057/4805-017 Braga/Guimarães, Portugal; 3 Stemmatters, Biotecnologia e Medicina Regenerativa SA, 4805-017 Guimarães, Portugal; rasousa@stemmatters.com

**Keywords:** Parkinson’s disease, aging, 6-hydroxydopamine, animal model, neurodegeneration

## Abstract

Parkinson’s disease (PD) is the second most common age-related neurodegenerative disorder. The neurodegeneration leading to incapacitating motor abnormalities mainly occurs in the nigrostriatal pathway due to the loss of dopaminergic neurons in the substantia nigra pars compacta (SNpc). Several animal models have been developed not only to better understand the mechanisms underlying neurodegeneration but also to test the potential of emerging disease-modifying therapies. However, despite aging being the main risk factor for developing idiopathic PD, most of the studies do not use aged animals. Therefore, this study aimed at assessing the effect of aging in the unilateral 6-hydroxydopamine (6-OHDA)-induced animal model of PD. For this, female young adult and aged rats received a unilateral injection of 6-OHDA into the medial forebrain bundle. Subsequently, the impact of aging on 6-OHDA-induced effects on animal welfare, motor performance, and nigrostriatal integrity were assessed. The results showed that aging had a negative impact on animal welfare after surgery. Furthermore, 6-OHDA-induced impairments on skilled motor function were significantly higher in aged rats when compared with their younger counterparts. Nigrostriatal histological analysis further revealed an increased 6-OHDA-induced dopaminergic cell loss in the SNpc of aged animals when compared to young animals. Overall, our results demonstrate a higher susceptibility of aged animals to 6-OHDA toxic insult.

## 1. Introduction

Parkinson’s disease (PD) is the second most prevalent neurodegenerative disease worldwide. Its global prevalence is estimated to be 0.3%, increasing with aging to more than 3% in individuals over 80 years of age [1]. Clinically, PD is mainly characterized by a core of specific motor disabilities, such as tremor, rigidity, postural instability, and bradykinesia, which are primarily caused by the progressive degeneration of dopamine-containing neurons (DA neurons) in the substantia nigra pars compacta (SNpc) and the consequent decline of the striatal DA content [2]. Intracytoplasmic inclusions of aggregated α-synuclein (Lewy bodies) are a typical feature of the disease, in which mechanisms associated to mitochondrial dysfunction, oxidative stress, and neuroinflammation also play an important role in the aggregation process and the neurodegeneration caused by it [3]. Commonly, PD is an idiopathic disease and only a few cases (5%–10%) are associated with PD-linked genes, known as familial forms of PD. Yet, idiopathic and genetic forms contain similar mechanisms involved in the progression of the disease [4]. In fact, the etiology of PD is multifactorial. Factors, such as genetic background, environmental agents, as well as aging, seem to contribute to the development and progression of this neurodegenerative disorder [5]. From these, aging represents the greatest risk factor for PD, as noted by the sharp increase in PD prevalence with aging [1,6]. Indeed, it is largely recognized that aging affects several neurochemical properties of the dopaminergic system, including changes in dopamine synthesis, metabolism, uptake, and receptor sensitivity, which makes neurons of the nigrostriatal pathway preferentially vulnerable to deal with aging compared to other regions of the central nervous system [7]. Moreover, these age-related changes have been reported to increase the dopaminergic system’s vulnerability to neurotoxic insults/toxins [8,9,10].

Unfortunately, there is still no cure or neuroprotective treatment for PD [11,12] and efforts have been made to develop alternative and disease-modifying therapeutic approaches [13,14,15,16,17]. For this, the use of pre-clinical animal models of PD is a crucial step to develop and screen new therapeutic strategies. Current animal models of PD include chemically induced models through the administration of environmental or synthetic agents and genetic models expressing PD-associated mutations or truncated proteins like alpha-synuclein [18,19]. 6-hydroxydopamine (6-OHDA) neurotoxin is extensively used to lesion the nigrostriatal dopaminergic system as a model of PD. Although the exact mechanism by which 6-OHDA causes neurodegeneration is not fully understood, it is known that is a dopamine analogue that targets DA transporter to access the cytosol of DA neurons, where it generates mitochondrial respiratory dysfunction, leading to high levels of oxidative stress and subsequent neuronal dysfunction and death within the nigrostriatal pathway [19].

Still, although aging is well recognized as an important risk factor for PD, most of the animal models of disease, including the ones induced by toxins, such as 6-OHDA, use male young rodents. Indeed, only a few studies have evaluated the effect of aging on their neuropathology and behavior outcomes [20,21,22,23,24,25]. This could compromise the face validity of these animal models, particularly concerning therapeutic screening studies. Having this in mind, this work aimed at comparing young and aging female rats following unilateral 6-OHDA/vehicle injection into the medial forebrain bundle (MFB). To look for age-related changes in this animal model, motor function, including apomorphine-induced rotation behavior, the symmetry of spontaneous forelimb use on the cylinder apparatus, and fine motor skills, was assessed at different time points after lesion. Moreover, body weight variation throughout the experiment was assessed as a surrogate parameter of animal welfare. Finally, the extension of dopaminergic degeneration within the striatum and SNpc was evaluated at the end of the experiment. With this study, we demonstrated that aging had a negative impact on animal welfare after surgery both in lesioned and non-lesioned rats. Contrarily, aging *per se* did not affect either motor performance or DAergic neuronal assessment in the striatum and SNpc. Moreover, age did not have a significant impact on toxin-induced rotation behavior and the degree of forelimb usage asymmetry during animal spontaneous exploration of the cylinder. Yet, 6-OHDA insult affected significantly more the skilled motor function of aged rats, which might be related to a higher degree of dopaminergic degeneration in the SNpc observed in aged lesioned animals when compared with their younger counterparts.

## 2. Results

### 2.1. Animal Welfare Varied among Experimental Groups

**Figure 1:**
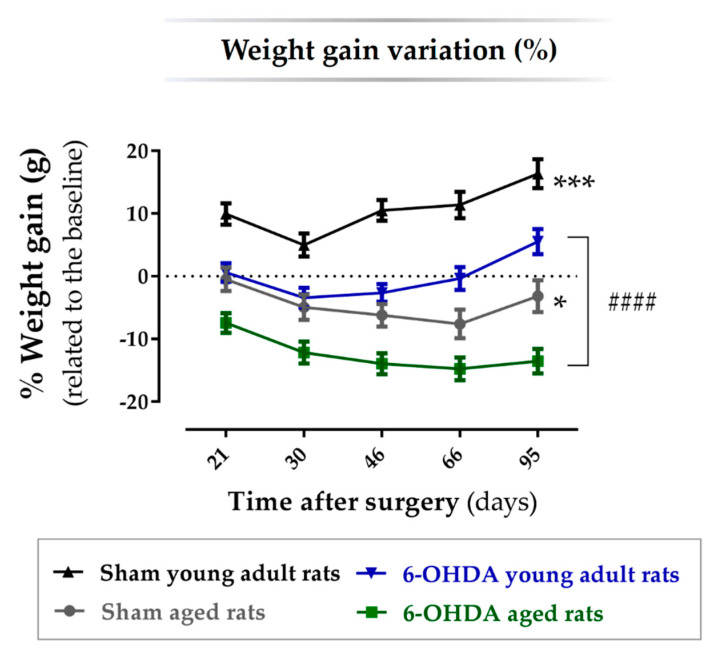
Percentage of body weight variation related to baseline at 21-, 30-, 46-, 66-, and 96-days post-surgery. The weight variation throughout the time of the experiment is statistically different between groups. Both young adult and aged 6-hydroxidopamine (6-OHDA)-lesioned animals had a significantly lower weight gain percentage along the experiment when compared with respective sham animals. Moreover, aged 6-OHDA-lesioned animals had a significantly lower weight gain when compared with young lesioned animals. *n* = 6 for the sham young adult rats group, *n* = 8 for the 6-OHDA young adult rats group, *n* = 5 for the sham aged rats group and *n* = 9 for the 6-OHDA aged rats group. Data are presented as mean ± SEM for each timepoint. #### *p* ≤ 0.0001 for the statistical difference between aged and adult young 6-OHDA-lesioned rats; * *p* ≤ 0.05, *** *p* ≤ 0.001 for sham animals statistically different from respective 6-OHDA groups. Abbreviations: 6-OHDA, 6-hydroxidopamine.

Animals were divided into four groups: young adult rats (*n* = 8; 10 weeks old; 6-OHDA young adult rats group) and aged rats (*n*= 9; 17 months old; 6-OHDA aged rats group) receiving unilateral 6-OHDA injection into the MFB, and young adult rats (*n* = 6; 10 weeks old; Sham young adult rats group) and aged rats (*n*= 5; 17 months old; Sham aged rats group) receiving vehicle infusion, used as controls for each lesioned group. Three animals of the 6-OHDA aged rats group and one animal of the Sham aged rats group died prematurely after surgery and were not considered. Contrarily, no young animal died after surgery. The aged animal groups also showed a delay when waking up from anesthesia and in the resumption of motor activity after surgery, when compared with the young adult animal groups. Yet, both young and aged animals that survived to surgery had no lasting post-operative complications. The body weight variation throughout the experiments is regarded as a surrogate parameter for animal welfare. Therefore, the body weight of each animal was assessed before surgery (baseline) and 21, 30, 46, 66, and 95 days after. The percentage change in body weight at these time points was calculated concerning the weight measured before lesion surgery (baseline). The results showed a significant main effect for the factor treatment, i.e., vehicle vs. 6-OHDA (F(1, 24) = 30.8, *p* ≤ 0.0001, η^2^_partial_ = 0.56) and for the factor age, i.e., 10 weeks old vs. 17 months old (F(1, 24) = 67.2, *p* ≤ 0.0001, η^2^_partial_ = 0.74), but no interaction effect between the factors treatment and age. A significant main effect for the factor time (days; F(2.46, 7.57) = 31.9, *p* ≤ 0.0001, η^2^_partial_ = 0.57) and an interaction effect between the factors time and age was also observed (F(2.46, 7.57) = 38.3, *p* ≤ 0.0001, η^2^_partial_ = 0.54). As shown in Figure 1, the group of aged animals lesioned with 6-OHDA lost more weight throughout the experiment, followed by aged animals injected with the vehicle. Young adult animals injected with the vehicle did not lose weight along the experiments (%weight gain > 0), while animals of the same age but injected with 6-OHDA lost some weight but then recovered it throughout the experiment. After performing a pairwise comparison between groups, the results showed that the percentage of weight gain along the experiment was significantly different in both aged (*n* = 9, *p* < 0.05; Table 1) and young (*n* = 8, *p* < 0.001; Table 1) 6-OHDA-lesioned animals, when compared with the respective sham groups (*n* = 5 for the sham aged rats group and *n* = 6 for the sham young adult rats group). Moreover, the percentage of weight gain was also significantly different between the aged and young lesioned animals (*p* < 0.0001; Table 1).

### 2.2. Aging Does Not Affect the Impact of the Lesion on the Turning Behavior and Degree of Forelimb Use Asymmetry

**Figure 2:**
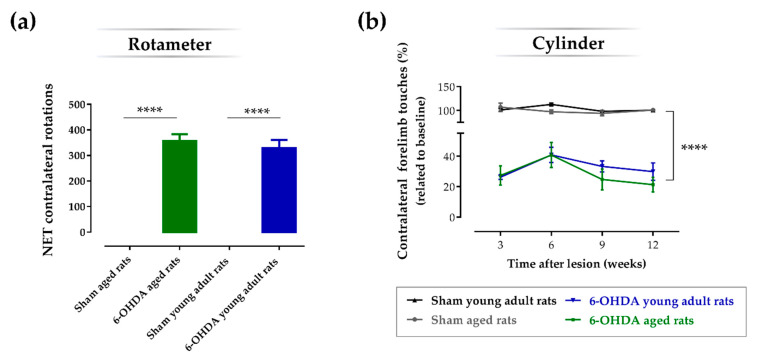
Apomorphine-induced turning behavior (rotameter test) and spontaneous contralateral forelimb use (cylinder test). The rotameter test (**a**) revealed that both aged and young adult animals injected with 6-hydroxidopamine (6-OHDA) exhibited an intense turning behavior when compared to respective sham groups. No significant differences were observed on turning behavior between aged and young adult rats. The cylinder test (**b**) revealed that both aged and young adult animals injected with 6-OHDA used the contralateral forelimb significantly less after lesion when compared to the respective sham groups. No significant differences were observed between aged and young adult animals regarding forelimb use asymmetry. For rotameter: *n* = 6 for the sham young adult rats group; *n* = 8 for the 6-OHDA young adult rats group; *n* = 5 for the sham aged rats group; and *n* = 8 for the 6-OHDA aged rats group. For the cylinder test: *n* = 5 for the sham young adult rats group, *n* = 5 for the 6-OHDA young adult rats group, *n* = 5 for the sham aged rats group, and *n* = 6 for the 6-OHDA aged rats group. Data are presented as mean ± SEM. **** *p* ≤ 0.0001: Sham animals were statistically different from respective 6-OHDA groups. Abbreviations: 6-OHDA, 6-hydroxidopamine.

The apomorphine-induced turning behavior test (or rotameter test) was performed to evaluate the functional integrity of the DAergic system after 6-OHDA injection, and to select the animals that were truly injured 3 weeks after lesion (Figure 2a). Analysis of variance revealed statistical differences in the number of apomorphine-induced rotations between groups (F(3, 23) = 59.0, *p* ≤ 0.0001, η2 = 0.89). Post-hoc analyses using the Sidak test indicated that both aged (356 ± 27.5; *n* = 8; *p* ≤ 0.0001) and young adult (329 ± 31.9; *n* = 8; *p* ≤ 0.0001) animals lesioned with 6-OHDA performed significantly more apomorphine-induced rotations when compared with respective sham animals of the same age (0 ± 0; *n* = 5 for the sham aged rats group; 0 ± 0; *n* = 6 for the sham young adult rats group). No significant differences in the number of apomorphine-induced rotations were observed between the aged and young adult lesioned groups (*p* > 0.05). The cylinder test measures the symmetry of animal forelimb usage during the exploration of an unknown vertical cylinder wall. Weight-bearing wall touches using contralateral and ipsilateral forelimbs were counted to evaluate the limb use asymmetry after 6-OHDA unilateral lesion. The results showed a significant main effect for the factor treatment, i.e., vehicle vs. 6-OHDA (F(1, 17) = 421, *p* ≤ 0.0001, η^2^_partial_ = 0.96) but no main effect for the factor age, i.e., 10 weeks old vs. 17 months old, was observed (F(1, 17) = 1.16, *p* = 0.30, η^2^_partial_ = 0.06). When a pairwise comparison between groups was performed, the results showed that both aged (*n* = 6; *p* ≤ 0.0001) and young adult (*n* = 5; *p* ≤ 0.0001) 6-OHDA-lesioned animals performed significantly less touches with the contralateral forelimb (lesioned forelimb) when compared with their sham counterparts, respectively (*n* = 5 for both sham groups). However, no significant differences were observed between aged and young adult groups lesioned with 6-OHDA (*p* > 0.05) regarding the percentage of touches using the contralateral forelimb (Figure 2b; Table 2). Statistical analyses also showed a significant main effect for the factor time (weeks; F(3, 51) = 4.61, *p* ≤ 0.01, η^2^_partial_ = 0.21), since the number of contralateral forelimb touches of the lesioned groups significantly decreased in week 9 (*p* < 0.05) and 12 (*p* < 0.05), when compared with week 6. 

### 2.3. Aging Affects the Impact of Lesions on Skilled Motor Function

**Figure 3:**
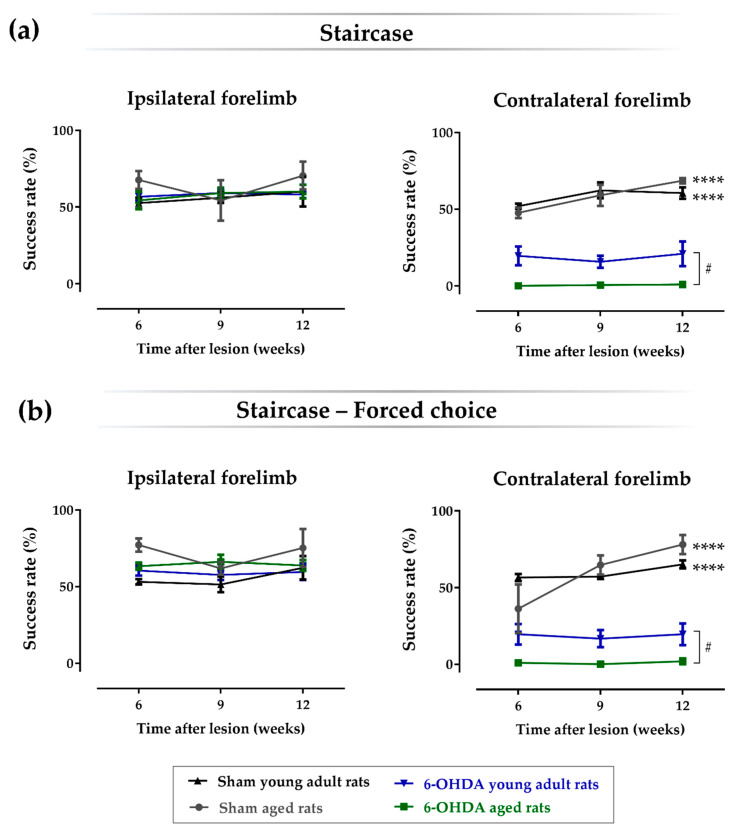
Staircase test. Both aged and young adult 6-hydroxidopamine (6-OHDA)-injected animals presented a significant impairment in the paw-reaching test performance regarding the contralateral forelimb when compared with the respective sham groups in both the double sides (**a**) and forced-choice task (**b**). Compared with 6-OHDA-lesioned young adult rats, paw reaching ability using the contralateral forelimb was more affected in lesioned aged animals for both the double sides (**a**) and forced-choice tasks (**b**). No differences between groups were observed in the performance of the ipsilateral forelimb for this behavioral test between all experimental groups. Furthermore, age *per se* did not have an impact on the skilled motor function of rats, since no significant differences were observed between young adult and aged sham animals for both the ipsi- and contralateral forelimbs. *n* = 5 for the sham young adult rats group, *n* = 5 for the 6-OHDA young adult rats group, *n* = 3 for the sham aged rats’ group, and *n* = 6 for the 6-OHDA aged rats group. Data are presented as mean ± SEM. # *p* ≤ 0.05 for the statistical difference between aged and adult young 6-OHDA-lesioned rats; **** *p* ≤ 0.0001 for sham animals statistically different from the respective 6-OHDA groups. Abbreviations: 6-OHDA, 6-hydroxidopamine.

The staircase test was performed to assess the forelimb’s fine movements, namely the animal’s ability to reach, grasp, and perform paw-to-mouth movement to eat food pellets, which are positioned on both the left and right sides along a staircase containing several steps of different reaching difficulty. As shown in Figure 3, the results regarding the success rate for eaten pellets using the contralateral paw (lesioned forelimb) showed a significant main effect for the factor treatment, i.e., vehicle vs. 6-OHDA (F(1, 16) = 145, *p* ≤ 0.0001, η^2^_partial_ = 0.90), factor age (F(1, 16) = 5.02, *p* = 0.040, η^2^_partial_ = 0.24), and the interaction between these two factors (F(1, 16) = 5.16, *p* = 0.037, η^2^_partial_ = 0.24). Statistical analyses also showed a significant main effect for the factor time (weeks; F(2, 32) = 7.66, *p* ≤ 0.01, η^2^_partial_ = 0.32) and the interaction between time and treatment (F(2, 32) = 6.82, *p* ≤ 0.01, η^2^_partial_ = 0.30). When a pairwise comparison between groups was performed (Table 3), the results demonstrated that both aged (*n* = 6; *p* ≤ 0.0001) and young adult (*n* = 5; *p* ≤ 0.0001) animals lesioned with 6-OHDA showed a significant decrease on the success rate of eaten pellets with the contralateral forelimb when compared with respective sham animals of the same age (*n* = 3 for the sham aged rats group, and *n* = 5 for the sham young adult rats group). Moreover, skilled motor function of the contralateral forelimb was significantly more affected on aged animals lesioned with 6-OHDA when compared with the young adult counterparts (*p* < 0.05). As expected, no differences were observed regarding the success rate of eaten pellets with the ipsilateral forelimb between all groups. Similar results were obtained in the forced-choice task, in which the pellets are placed only at one side of the staircase (left or right). A significant main effect was observed for the factor treatment (F(1, 16) = 148, *p* ≤ 0.0001, η^2^_partial_ = 0.90), factor age (F(1, 16) = 4.60, *p* = 0.048, η^2^_partial_ = 0.22), and the interaction between these two factors (F(1, 16) = 4.66, *p* = 0.046, η^2^_partial_ = 0.23). Statistical analyses also showed a significant main effect for the factor time (weeks; F(1.20, 19.2) = 10.0, *p* ≤ 0.01, η^2^_partial_ = 0.38) and the interaction between time and treatment (F(1.20, 19.2) = 9.6, *p* ≤ 0.01, η^2^_partial_ = 0.38), time and age (F(1.20, 19.2) = 5.3, *p* ≤ 0.05, η^2^_partial_ = 0.25), and between the three factors (F(1.20, 19.2) = 5.3, *p* ≤ 0.05, η^2^_partial_ = 0.25). Accordingly, pairwise comparison between the groups (Table 3) showed that for the forced choice to the contralateral forelimb, both aged (*n* = 6; *p* ≤ 0.0001) and young adult (*n* = 5; *p* ≤ 0.0001) animals lesioned with 6-OHDA showed a significant decrease on the success rate of eaten pellets when compared with the respective sham animals of the same age (*n* = 3 for the sham aged rats group, and *n* = 5 for the sham young adult rats group). Consistently, for the forced-choice task, the skilled motor function of the contralateral forelimb was also significantly more affected in aged animals lesioned with 6-OHDA when compared with their young adult counterparts (*p* < 0.05). As expected, for the forced-choice task using the ipsilateral forelimb, no differences were observed regarding the success rate of eaten pellets. Moreover, the results also show that age itself does not have an impact on the skilled motor function of rats, since no significant differences were observed between young adult and aged sham animals for both the ipsi- and contralateral forelimbs (*p* > 0.05).

### 2.4. Aging Exacerbates Lesion-Induced Dopaminergic Degeneration within SNpc

**Figure 4:**
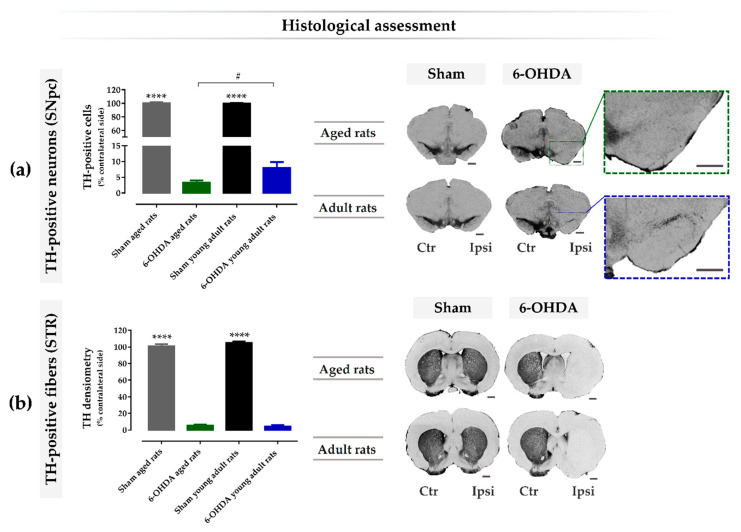
Histological assessment of 6-hydroxidopamine (6-OHDA)-induced nigrostriatal degeneration in young and aged animals. Quantification and representative micrographs of tyrosine hydroxylase (TH)-positive neurons and fibers in the substantia nigra pars compacta (SNpc) (**a**) and striatum (**b**), respectively. Results are presented as a percentage for the control side (non-lesioned hemisphere), showing a significant reduction of TH-expressing cells in 6-OHDA-lesioned animals in comparison to sham animals in the SNpc (**a**) and in the striatum (**b**). Multiple comparison analyses revealed a significatively more pronounced reduction in aged 6-OHDA-lesioned animals when compared with young adult lesioned animals in the SNpc (**a**). *n* = 6 for the sham young adult rats group, *n* = 8 for the 6-OHDA young adult rats group, *n* = 5 for the sham aged rats group, and *n* = 8–9 for the 6-OHDA aged rats group. Data are presented as mean ± SEM. # *p* ≤ 0.05 for the statistical difference between aged and adult young 6-OHDA-lesioned rats; **** *p* ≤ 0.0001 for sham animals statistically different from the respective 6-OHDA groups. Scale bar for (a) SNpc: 500 μm, and for (**b**) STR: 1mm. Abbreviations: CTR, contralateral; IPSI, ipsilateral; 6-OHDA, 6-hydroxidopamine; SNpc, substantia nigra pars compacta; TH, tyrosine hydroxylase; STR, Striatum.

To analyze the effects of the 6-OHDA lesion at the histological level, staining for tyrosine hydroxylase (TH) was performed in the SNpc and striatum. TH-positive cells were counted in the SNpc and TH-positive fibers were analyzed by densitometry in the striatum. Both histological analyses in the lesioned hemisphere were compared and presented as a percentage of the control side (non-lesioned hemisphere). From the results, the analysis of variance revealed statistical differences in the number of TH-positive neurons in the SNpc between groups, 14 weeks after lesion (F(3, 27) = 1568, *p* ≤ 0.0001, η^2^ = 0.99; Figure 4a), as well as in the TH densitometry in the striatum (F(3, 27) = 1082, *p* ≤ 0.0001, η^2^ = 0.99; Figure 4b). Through multiple comparison analyses using the Sidak test, a significant decrease of DA neurons after the injection of 6-OHDA into the MFB was observed in both aged (3.25 ± 0.72; *n* = 9; *p* ≤ 0.0001) and young adult (*n* = 7.93 ± 1.86; *n* = 8; *p* ≤ 0.0001) animals, when compared with the respective vehicle controls (100 ± 1.54, *n* = 5 for the sham aged rats group; and 99.7 ± 0.92, *n* = 6 for the sham young adult rats group; Figure 4a). Consistently, striatal TH-positive fibers were also significantly decreased after 6-OHDA lesion in both aged (5.50 ± 1.35; *n* = 8; *p* ≤ 0.0001) and young adult rats (4.29 ± 1.52; *n* = 9; *p* ≤ 0.0001), when compared with the respective controls (101 ± 2.31, *n* = 5 for the sham aged rats group; and 105 ± 1.79, *n* = 6 for the sham young adult rats group; Figure 4b). Of note, the decrease in TH-positive neurons within the SNpc was significantly greater in lesioned aged animals when compared to the younger ones (*p* < 0.05, Figure 4a); however, there was no difference regarding the striatal TH densitometry between these groups (*p* > 0.05, Figure 4b).

## 3. Discussion

Aging is well recognized as the biggest risk factor for developing idiopathic PD [6,7]. However, the majority of pre-clinical studies using PD animal models disregard the aging factor, most often using young adult animals. Therefore, with this study, we aimed at assessing the effect of age in one of the most used animal models of PD, which is induced by a 6-OHDA unilateral lesion into the MFB of rats. This is a well-defined rat model of PD that leads to degeneration of DA neurons in the nigrostriatal pathway and consequently mimics the presence of the main motor deficits observed in the disease [13,14,26]. Contrarily to lesions in the SNpc and striatum, MFB lesions have the advantage of mimicking the late stage of PD [27]. Moreover, it avoids direct mechanical damage of the SNpc and striatum, allowing further screening studies, such as the injection of cell grafts, neuroprotective molecules, as wells as the cellular secretome into these brain regions [13,14,16,28].

**Figure 5:**
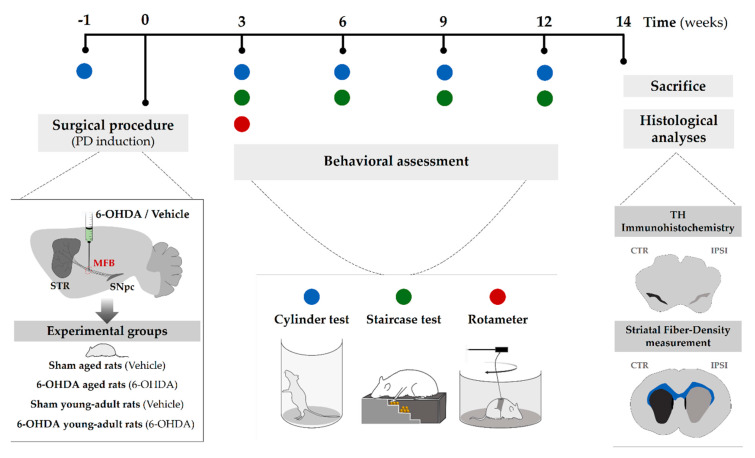
Experimental design: After baseline testing for the cylinder test, the Parkinson’s disease (PD) model was induced by a 6-hydroxidopamine (6-OHDA) unilateral injection into medial forebrain bundle (MFB) in the right hemisphere. Three weeks later, the animals were submitted to a first behavioral analysis (using the cylinder, staircase, and rotameter tests) to characterize the model. The cylinder and staircase tests were repeated 6, 9, and 12 weeks after surgery. Lastly, the animals were sacrificed for further histological analysis. Abbreviations: 6-OHDA, 6-hydroxidopamine; MFB, medial forebrain bundle; PD, Parkinson’s disease; SNpc, substantia nigra pars compacta; STR, striatum; TH, tyrosine hydroxylase.

To study the effect of age in this model, we used female rats lesioned with 6-OHDA in the right MFB of different ages, namely 10 weeks old and 17 months old, whose ages correspond to adult young and aged animals, respectively (Figure 5). As shown in the rotameter test (Figure 2a), both young and aged 6-OHDA-injected animals exhibited an intense contralateral turning behavior three weeks after lesion when compared with the respective sham groups, which indicates an evident decay in the functional integrity of the dopaminergic system in the ipsilateral brain hemisphere. Moreover, 6-OHDA-induced dopaminergic damage in the right hemisphere was also confirmed with the cylinder test, in which both lesioned groups displayed contralateral motor deficits, as observed by the significant decrease in the number of touches using the contralateral forelimb in the cylinder wall over time when compared with the respective control groups (Figure 2b). No significant differences in contralateral motor deficits were observed in the cylinder test between young adult and aged animals. Consistently, the 6-OHDA lesion also affected fine motor skills over time, addressed by the staircase test, in both lesioned animal groups, as seen by the decrease in the success rate of eaten pellets using the contralateral paw, when compared with the respective vehicle controls (Figure 3). However, contrarily to the cylinder test, herein, age affected the impact of the lesion on the skilled motor function, as observed by the significant biggest decline in the success rate of eaten pellets using the contralateral paw by aged Parkinsonian animals, when compared with their younger counterparts (Figure 3). In addition to this, 6-OHDA injection into MFB also led to a significant decrease in TH-positive neurons in the ipsilateral SNpc when compared with the contralateral hemisphere in both the aged and young adult lesioned groups (Figure 4a). Consistently, a significant decline in the number of TH-positive fibers in the ipsilateral striatum was also observed by densitometry analyses in both lesioned groups, when compared with the contralateral striatum (Figure 4b). Note that aged 6-OHDA-lesioned animals lost significantly more DA neurons in the ipsilateral SNpc when compared with younger lesioned animals (Figure 4a). However, no differences were observed between these two groups regarding the striatal TH densitometry (Figure 4b). In fact, a time study using 6-OHDA-lesioned rodents into MFB demonstrated that a loss of TH-positive fibers in the striatum was fully established after just 1 week post-lesion and did not increase any further over time, contrarily to DA neurons in the substantia nigra (SN), the degeneration of which continued to progress [29]. This study suggests that the degeneration of TH fibers in the striatum occurs earlier than the loss of cell bodies within the SNpc in the 6-OHDA-lesioned model in the MFB, which could justify the lack of differences in the striatal TH densitometry found 14 weeks after lesion, while differences in the TH-positive cells number were still observed. The most pronounced loss in the number of DA neurons in the SNpc seen in older Parkinsonian animals, when compared with younger ones, could have mediated the worsening performance observed on the staircase test, which reflects a decline of the skilled motor function of aged animals (Figure 3).

Indeed, several data suggest that motor performance tends to decline with age [30,31]. Herein, we did not see any differences between aged and young sham rats regarding motor performance, but we showed that in toxin-lesioned rats, age harmed skilled motor function assessed with the staircase test. Yet, no differences were observed in the apomorphine-induced rotation behavior and the cylinder test between aged and young lesioned animals. However, it should be noted that the cylinder test provides a measure of spontaneous forelimb use, which is a low-complexity task when compared with the staircase test. Accordingly, studies demonstrated that the learning curve of younger and older adults is very similar in low-complexity tasks, whereas age-related alterations are statistically more pronounced in complex tasks [32]. Moreover, the staircase test proved to be highly sensitive to even mild DA-depleting lesions in rats. Even in partial DA-denervating lesions that did not result in animal apomorphine-induced rotation behavior, a significant impairment in the staircase test was observed [33]. This highlights the sensitivity of this test even when slight differences in dopaminergic lesions are present.

The literature has also been extensively reviewed about the particular vulnerability of the dopaminergic system to the aging process. Age-related changes on dopamine synthesis, metabolism, uptake, and receptor sensitivity, as well as disruption of the key mitochondrial processes, alterations on calcium dynamics, iron concentration, proteostatic dysfunction, and telomere shortening, are some of the alterations that make neurons of the nigrostriatal pathway preferentially vulnerable to death during aging [7,34,35,36]. Moreover, the sensitivity of the nervous system to neurotoxic insults is also increased with aging [8]. Concomitant with this, our present study showed that aging exacerbates the 6-OHDA-induced neurodegeneration in the SNpc. Similar findings have already been reported by other studies that did reveal an increased susceptibility to different neurotoxins in aged animals. For instance, when compared with young mice, aged animals displayed a higher degree of dopaminergic degeneration caused by 1-methyl-4-phenyl-1,2,3,6-tetrahydropyridine (MPTP) toxin [37,38]. On the other hand, MPTP did not cause a significant decline in the number of TH neurons in the SNpc of aged monkeys, when compared with younger primates, but caused a significant decline of TH-positive fibers in the striatum, as well as a decrease in the soma size and optical density of TH in the SN [39]. In rotenone-induced rat models of PD, age-induced alterations were also observed, including a reduced striatal dopamine content, reduction in glutathione, and increased levels of malondialdehyde in the SNpc [40]; DA neuronal loss [9,41]; as well as motor behavior deficits [40,41]. Ultrastructural changes, such as the presence of swollen mitochondria in the striatum and robust lipofuscin deposits in the SNpc, were also seen in aged rotenone-lesioned rats [41]. Regarding age comparative studies using 6-OHDA-induced rodent models, while some authors reported a more pronounced depletion of striatal dopamine levels and release [21,22], a higher decrease of striatal dopamine-evoked overflow [21], a higher degree of dopaminergic neuronal degeneration [25], and a decrease in locomotor outcomes in aged rats lesioned with 6-OHDA [22] when compared with younger counterparts, others did not observe a higher depletion in dopamine levels in aged lesioned animals [20], as well as no substantial differences in the degree of 6-OHDA-induced dopaminergic degeneration [23,24]. Yet, behavioral outcomes, namely locomotion and rearing activity, were more affected in older animals, which the authors hypothesized to be more related to aging-related neurochemical changes rather than the slight decrease observed in the number of DA neurons in aged rats [24].

In addition, it seems that younger animals display compensatory mechanisms after toxin injury that disappear with aging. An increase in endogenous neurotrophic factors after toxin insult is observed in young rodents [42,43] and primates [44] but not in their aged counterparts. For example, 6-OHDA lesion induced a compensatory increase in brain-derived neurotrophic factor (BDNF) and in glial cell line-derived neurotrophic factor (GDNF; factors known to be crucial for neuronal protection and survival) in the denervated striatum of younger lesioned rats 2 weeks post-lesion, but the same increase was not detected in elderly rats [43]. Additionally, a compensatory increase in striatal DA activity reflected by the increase in the homovanillic acid/DA ratio was present in young adult and middle-aged monkeys following MPTP insult but was totally absent in aged monkeys [39].

Moreover, several studies have suggested that glial cells might play a role in age-related increased vulnerability of the dopaminergic system to additional toxin damage by exacerbating the neuroinflammatory response [25,37,45,46]. For instance, the higher degree of dopaminergic degeneration caused by MPTP in aged mice was accompanied by a distinct microglial activation pattern. While in young animals, microglial activation was completely abolished 7 days after lesion, in aged animals, microglial activation surrounded dopaminergic neurons was intensified at 3–7 days after lesion and it was only decreased on day 14. This suggests that microglial activation in the SN could be altered with aging, contributing to a higher susceptibility of older mice to neurotoxicity induced by MPTP [37]. Gordon and colleagues also demonstrated that aged rats displayed an exaggerated astrocyte reactivity following unilateral 6-OHDA lesion in the MFB, as seen by the time-dependent induction of glial fibrillary acidic protein (GFAP, an astrocytic marker) in the striatum, which was higher and more persistent over time in aged animals [46]. In fact, it seems that aging leads to a proinflammatory and pro-oxidant state that may favor an exaggerated response to toxin-induced injury. Villar-Cheda and collaborators [25] showed that aged rats have an increased expression of the nicotinamide adenine dinucleotide phosphate (NADPH) oxidase complex subunit p47 in the SN, as well as increased levels of tumor necrosis factor-α and interleucine-1β, a proinflammatory cytokine known to increase the susceptibility of DA neurons to degeneration [47]. Moreover, 6-OHDA lesion induced a significantly higher number of interleucine-1β-labeled microglial cells in the SN of aged rats than in young rats, which was accompanied by a higher loss of nigral DA neurons [25].

Importantly, these age-related changes consequently alter the response to therapeutic approaches in toxin-induced animal models. For example, the extent of BDNF protection was less in aged rats lesioned with 6-OHDA, when compared with younger lesioned animals [48]. Similarly, treatment with astaxanthin, a natural compound, was shown to attenuate neurotoxicity in the MPTP-induced mouse model, and had lower efficacy in aged lesioned animals [49]. Aging also affected the response of lesioned animals to cell-based therapies by decreasing the survival of DA neurons grafted into the lesioned striatum of aged 6-OHDA-lesioned rats [50].

Although some differences between studies using toxin-induced animal models have been reported and discussed herein, they might be justified by variances in the animal species and strains used, different toxins and doses applied, distinct brain regions that were lesioned (e.g., SNpc, striatum, MFB, or intraventricular), as well as the time post-lesion at which the degree of dopaminergic neurodegeneration, biochemical parameters, and behavioral outcomes were assessed. Nevertheless, it seems evident that aging affects, at least in some degree, the histological, neurochemical, and behavioral outcomes of PD animal models. Concomitant with others, the present study showed aged-related changes in a toxin-induced animal model of PD, demonstrating that young animals are better able to compensate for the effects of dopaminergic cell loss, which leads to the differences observed in skilled motor function. This suggests that aging is a critical factor to consider during the development of novel therapeutics for PD. Using aged animals to model disease might increase the predictive validity of studies testing new therapeutic approaches. However, most researchers do not use aged animals to model disease due to the time it takes for animals to reach the required age, as well as the cost of housing these animals for a long period. Moreover, some animal models, namely the one induced by 6-OHDA, require surgery to induce the Parkinsonian features. Similar to what happens in elderly humans [51], previous studies have shown that aged rats are more sensitive to general anesthesia and do not recover as well as young rats [52]. Age-related differences in post-surgery recovery were also observed in this study, such as an age-induced delay in waking up from anesthesia and in the resumption of motor activity, as well as a higher death rate after surgery. Moreover, the weight gain variation along the experiment (Figure 1) was significantly lower in aged rats than in their younger counterparts. Note that these age-related changes in the post-surgery recovery could be problematic if another surgical procedure is needed for treatment. Considering the difficulties regarding the regular use of aged animals, a question arises whether the less severe phenotype of young animals might affect the results from this model sufficiently to compensate the drawbacks of using aged animals. It is important to mention that the 6-OHDA-induced animal model of PD used in this study is a severe model leading to an abrupt neuronal depletion and, consequently, pronounced motor deficits. In this way, we were not expecting to see robust differences between aged and young Parkinsonian animals. Yet, even in a such severe model, the aging factor was able to present a significant negative impact. Moreover, more relevant than the degree of impact that aging has on the toxin animal model *per se* is its effect on the response to therapies regarding screening studies for PD. As mentioned previously, other studies using aged 6-OHDA-lesioned animals have displayed reduced therapeutic responses compared to their younger counterparts. It should be noted that the main purpose of the use of older animals is precisely that they could better mimic the clinical features of idiopathic PD and the consequent response to therapeutic approaches. Although the use of older animals has disadvantages, a model that better represents the clinical pathology within the elderly population could bring benefits in the long term as it will be more easily translational, decreasing the risk of therapies failing during clinical studies. If we could improve the translation from “bench to bedside”, global resources and consequent costs might be saved. However, some factors should be taken into consideration, such as the impact of 6-OHDA injury on older animals regarding their welfare and recovery after surgery. To overcome these drawbacks, researchers should be careful when designing experiments using aged animal models, and consider, for instance, the use of mild and more neurodegenerative-progressive animal models for PD. A better strategy would be to perform the lesion surgery when animals are younger and evaluate the disease phenotype with aging, which might result in a decreased animal death rate, improved animal welfare, and reduced postoperative care, ultimately reducing the associated costs.

## 4. Materials and Methods

### 4.1. Animals

Animal experiments were conducted following the local regulations on animal care and experimentation (European Union Directive 2010/63/ EU), and with consent from the Portuguese national authority for animal research, Direção Geral de Alimentação e Veterinária (DGAV; ID: DGAV28421, Lisbon, Portugal) and Ethical Subcommittee in Life and Health Sciences (SECVS; project ID: SECVS-142/2017, January 2017, University of Minho, Braga, Portugal). To assess the effect of aging in the 6-OHDA-induced animal model of PD, young adult (10 weeks old) and aged (17 months old) Wistar–Han female rats were used (Charles River Laboratories, Saint-germain-nuelles, France). All animals were kept in an appropriate and similar cage (2 animals per cage) in the same room. Animals were maintained under a controlled environment (temperature (22–24 °C), light (12 hours light/dark cycle), and humidity (55%)), with food and water ad libitum. Rats were handled 1 week before the beginning of experiments. All efforts were made to assure the welfare of the animals and to minimize the suffering and the number of animals used.

### 4.2. Experimental Design

The sequence of experiments carried out in this study is graphically outlined in Figure 5. Animals were assigned into four groups: young adult rats (*n* = 8; 10 weeks old; 6-OHDA young adult rats group) and aged rats (*n*= 9; 17 months old; 6-OHDA aged rats group) receiving unilateral 6-OHDA in the MFB, and young adult rats (*n* = 6; 10 weeks old; sham young adult rats group) and aged rats (*n*= 5; 17 months old; sham aged rats group) receiving vehicle infusion, used as controls for the respective lesioned groups. Firstly, all rats were subjected to one session of the cylinder test to obtain a pre-lesion baseline control to compare with sessions following the 6-OHDA lesion. After baseline testing for the cylinder test, animals underwent surgical procedures for either 6-OHDA or drug vehicle injection into MFB. Three weeks after surgery, the degree of the lesion was assessed in a series of motor behavioral tests, namely, the cylinder test, staircase test, and apomorphine-induced rotation test. Cylinder and staircase tests were repeated 6, 9, and 12 weeks after lesion surgery to evaluate the progression of the lesion along time. At the end of the behavioral assessment, animals were euthanized, and brains dissected and processed for TH histological analysis in the SNpc and striatum.

### 4.3. Surgical Procedure (6-Hydroxidopamine Lesion)

Before the surgical procedure, animals were intraperitoneally anesthetized with ketamine-medetomidine (75 and 0.5 mg/kg, respectively). Subsequently, both young adult and aged rats were positioned on a stereotaxic frame (Stoelting Co., Wood Dale, IL, USA) and unilaterally injected in the right hemisphere with 2 µL of 6-OHDA hydrochloride (H4381; Sigma-Aldrich, St. Louis, MO, USA) using a 30-gauge needle Hamilton syringe (Hamilton Bonaduz AG., Bonaduz, Switzerland). 6-OHDA was administrated at a rate of 1.0 µL/min at a concentration of 4 µg/µL dissolved in 0.9% NaCl with 0.2 mg/mL of ascorbic acid (A1968; Sigma-Aldrich, St. Louis, MO, USA). The neurotoxin was injected into the MFB (coordinates related to Bregma: AP= −4.4 mm, ML= −1.0 mm, DV= −7.8 mm), according to Paxinos and Watson [53]. After each injection, the needle was raised 2 mm and left there for 2 min to avoid the backflow of the needle tract. The other two groups of animals at the same ages were injected at the same conditions with the drug vehicle (0.2 mg/mL of ascorbic acid in 0.9% NaCl). During surgery, the eyes of the animals were protected from the light with Vaseline®. After surgery, the surgical region was cleaned with chlorhexidine (Sigma-Aldrich, St. Louis, MO, USA) and sutured with Surgiquick suture (Sutures Ltd, Wales, United Kingdom). Animals were subcutaneously injected with an antibiotic Enrofloxacin/ Baytril® (5 mg/kg; Bayer Corporation, Whippany, NJ, USA), analgesic Butorphanol/Butomidor® (5 mg/kg; Richter Pharma AG, Wels, Austria), and anesthetic antagonist Antisedan®/Atipamezole hydrochloride (1 mg/kg; Pfizer Inc., Brooklyn, NY, USA). Then, rats were placed comfortably in a heated cage with food easily available to allow their recovery. Animals were carefully monitored during the following days. 

### 4.4. Animal Welfare—Body Weight Measurement

The body weight of each animal was measured before surgical procedure, as well as 21, 30, 46, 66, and 95 days after lesion. The percentage change in body weight at these time points was calculated concerning the initial weight as an overall indicator of animal welfare. 

### 4.5. Behavioral Assessment

#### 4.5.1. Apomorphine-Induced Rotation Test

As previously described for our group [13,26], the apomorphine-induced rotation test (also named rotameter) was performed three weeks after the 6-OHDA lesion to evaluate the degree of dopaminergic depletion. 6-OHDA lesion results in nigrostriatal denervation and subsequent hypersensitivity of the DA receptors [19]. Administration of apomorphine, a strong DA agonist, in lesioned-side animals generates an imbalance in the striatal dopaminergic transmission, preferentially stimulating the affected side. This unequal stimulation leads to the contralateral rotation of the animals. For this test, rats were subcutaneously administrated in the neck with 0.05 mg/kg apomorphine hydrochloride (Sigma-Aldrich, St. Louis, MO, USA) dissolved in 0.9% NaCl. Total net contralateral rotations (which represents the difference between the total number of contralateral and ipsilateral rotations) were quantified using an automated metal testing bowl (Med Associates Inc., Albany County, NY, USA) over a 45-min session. Since apomorphine is a strong DA agonist, continuous overstimulation of the dopaminergic system could result in an inadequate interpretation of the other outcomes evaluated in this study. Thus, this test was only performed once to select the animals that were injured after 6-OHDA lesions, i.e., presenting more than 100 contralateral rotations. 6-OHDA-lesioned animals performing less than 100 contralateral rotations were excluded from the study.

#### 4.5.2. Cylinder Test

The cylinder test, originally described by Schallert and Tillerson (2000) [54], is a drug-free sensorimotor behavioral test commonly used in rodents to assess the limb use asymmetry after 6-OHDA unilateral lesion [16,55]. To perform this test, rats were placed individually in a glass and transparent cylinder (21 cm diameter, 34 cm height) and were videotaped for 5 min to record the spontaneous forelimb use of the animals when exploring the cylinder wall. A mirror was placed behind the cylinder at an angle that allowed the recording of the forelimb movements along 360°. The videos were analyzed in slow motion and frame by frame to score the movements over the 5 min. The number of supporting wall contacts that the animal performed with the right and the left forelimb during rearing and lateral movements along the wall were quantified. When the rat explored the cylinder wall with a single forelimb, it was recorded as an independent wall placement for that limb. The first forelimb to contact the wall was also scored as an independent wall placement for that limb. Placement of the other forelimb on the wall while keeping the initial placement was scored as a simultaneous movement for that limb. If the animal simultaneously placed both forelimbs on the wall, it was scored as co-use of both limbs. In the end, the performance of the contralateral forelimb (sum of independent and simultaneous touches in the cylinder wall using the contralateral forelimb) was calculated as a percentage of the total performance (sum of the independent and simultaneous touches to both left and right forelimbs). The pre-lesion cylinder test was conducted in one session as a baseline analysis for comparison with sessions following 6-OHDA lesion and to validate the model. This test was performed during the night cycle when animals are more active in exploration, and all trials were performed at the same time of the day. Animals performing less than 40 touches on the wall were eliminated from the analysis. 

#### 4.5.3. Staircase Test

The staircase test was performed to assess animals’ forelimb fine movements, namely animals’ ability to reach, grasp, and perform the paw-to-mouth movement to eat the pellets as previously described [56,57]. For this, animals were placed in a staircase box (Campden Instruments Ltd., Lafayette, IN, USA). The staircase apparatus consists of a clear chamber with an articulated lid. This chamber has a thin compartment with a central podium along its extent. A removable double stair of 7 steps on each side is docked on the sides of this thin compartment containing 5 pellets on each step. The test has a duration of 8 days. On the first day, animals were kept inside the box for 10 min to become familiarized with the staircase apparatus. In the following 5 days, each animal was kept inside the box for 15 min to grasp, retrieve, and eat the pellets along the seven graded stages of reaching difficulty. In the end, the number of pellets that were eaten and lost on both sides was recorded. In the last 2 days, the motor impairment of the affected side was assessed through a forced-choice task, in which the pellets were only placed on one of the step sides (left or right). It should be noted that all trials were performed at the same time of the day. During the experiment, animals’ access to food was restricted. Since the performance of the staircase test depends not only on the animals’ motor skills but also on their learning capability and motivation, animals that did not eat pellets on either the left or right side of the staircase were excluded from the analyses.

### 4.6. Histological Assessment

After behavioral assessment (14 weeks after surgery), animals were deeply anesthetized with sodium pentobarbital/Eutasil® (60 mg/kg; Ceva Santé Animale SA., Libourne, France) by intraperitoneal injection and transcardially perfused using 0.9% NaCl followed by 4% paraformaldehyde (PFA; Merck & Co. Inc., Kenilworth, NJ, USA) dissolved in 0.01 M phosphate-buffered saline (PBS). Rat brains were dissected and post-fixed in 4% PFA dissolved in 0.01 M PBS for 48 h, followed by immersion in a 30% sucrose solution dissolved in 0.01 M PBS. After sinking, the brains were sectioned into coronal slices (50 μm thick) using a vibratome (Leica Vibratome VT1000S; Leica Biosystems, Nußloch, Baden-Württemberg, Germany) and processed as free-floating sections.

#### 4.6.1. TH Immunohistochemistry

Four series of striatal and mesencephalon coronal sections were immersed in 3% of hydrogen peroxide dissolved in 0.01 M PBS for 20 min at room temperature (RT) to inhibit endogenous peroxidase activity, and then washed with 0.01 M PBS for 10 min and permeabilized with 0.3% PBS-Triton (PBS-T) 3 times for 10 min each at RT. After permeabilization, sections were blocked using 10% of fetal calf serum (FCS; Sigma-Aldrich, St. Louis, MO, USA) diluted in 0.01 M PBS for 2 h at RT. Afterwards, brain slices were incubated with rabbit TH primary antibody (1:1000; AB152-Merck Millipore, Burlington, MA, USA) diluted in 0.01 M PBS with 2% of FCS, overnight at 4 °C. On the following day, sections were rinsed 3 times with PBS-T for 10 min each and incubated with a biotinylated secondary antibody (TP-125-BN; ThermoFisher Scientific, Waltham, MA, USA) for 30 min at RT. The next steps were performed while avoiding exposure to the light. After washing 3 times with PBS-T for 10 min each, sections were incubated with streptavidin peroxidase solution (TS-125-HR; ThermoFisher Scientific, Waltham, MA, USA) for 30 min at RT. Then, slices were washed 3 times in PBS-T for 10 min each and incubated with 0.05 M Tris-HCl buffer (pH = 7.6) for 10 min at RT. For antigen visualization, sections were incubated with 3,3’- diaminobenzidine tetrahydrochloride (DAB; D5905; 0,5 mg/mL; Sigma-Aldrich, St. Louis, MO, USA) dissolved in 0.05 M Tris-HCl buffer with 0.025% of hydrogen peroxide for 5 min at RT. Reactions with DAB were stopped with the immersion of slices in Tris-HCl buffer and sections were then mounted on superfrost slides (ThermoFisher Scientific, Waltham, MA, USA). After drying in the dark, sections were mounted using Entellan® (ref. 107961.0500; Merck Millipore, Burlington, MA, USA). Using a brightfield microscope (model no. BX51; Olympus, Tokyo, Japan) equipped with a digital camera (PixeLINK PL-A622; CANIMPEX Enterprises, Halifax, NS, Canada) and using the Visiopharm integrator system software (version 2.12.3.0; Visiopharm, Hørsholm, Denmark), four identical TH-labeled slices covering the entire mesencephalon, including all the portions of the SNpc, were analyzed. The boundaries of the SNpc area were drawn through the identification of anatomic standard reference points with the help of the rat brain atlas [53]. Total TH-positive cells in the SNpc area were counted on both hemispheres. The data were presented as the percentage (%) of the remaining TH-positive cells in the lesioned side compared to the control side.

#### 4.6.2. Striatal Fiber-Density Measurement

Four identical TH-immunostained striatal sections were photographed using brightfield illumination (model no. SZX16; Olympus, Tokyo, Japan). All image analysis was performed using the Fiji software (64-bit version; NIH, Bethesda, MA, USA). The optical density of TH-positive fibers was measured by densitometry. Photos were converted to grayscale; black and white values were inverted. Using “polygon selections”, striatal areas of both the ipsi- and contralateral hemispheres were delineated and analyzed for the mean gray value, which represents the average gray value within the selection, i.e., the sum of the gray values of all the pixels in the selection divided by the number of pixels. The mean gray value of three different regions of the cortex (internal control) was also measured in both hemisphere sides. The average value obtained in the cortex was subtracted from the value of the respective striatum to eliminate the nonspecific background. The data are presented as the percentage (%) of the contralateral striatum (non-lesioned side).

### 4.7. Statistical Analysis

Data regarding the histological assessment and behavioral analysis of the apomorphine-induced rotation test were analyzed using one-way ANOVA to compare the mean values for the four groups. When an evaluation along time was required (weight gain variation, staircase and cylinder tests), a mixed design factorial ANOVA was performed. Pairwise comparisons between groups based on estimated marginal means using Sidak’s correction were performed. The significance value was set as *p* ≤ 0.05 for all statistical tests and all the results are presented as mean ± SEM (standard error of the mean). IBM SPSS^®^ Statistics (version 23.0; IBM Co., Armonk, NY, USA) was the statistical software platform used for the statistical analysis and GraphPad Prism (version 6.01/b; GraphPad Software, Inc., San Diego, CA, USA) was used to perform graphic representation.

## 5. Conclusions

Although aging is recognized as the biggest risk factor for developing idiopathic PD, most of pre-clinical studies use young adult animals. Having this in mind, this study aimed to assess whether a commonly used PD animal model, induced by a 6-OHDA unilateral lesion into the MFB of rats, disclosed behavioral or histological differences between aged and young animals. Our results showed a higher susceptibility of aged rats to surgery, demonstrating a slower recovery after surgery, higher animal death rate and impaired animal welfare along experiment. Moreover, aged animals displayed an increased 6-OHDA-induced dopaminergic cell loss in the SNpc, which led to a worst motor performance regarding skilled motor function. Overall, the present study demonstrated age-related changes in a toxin-induced animal model of PD, suggesting that aging should be considered in pre-clinical studies, which might be fundamental to increase the predictive validity of therapeutic approaches for idiopathic PD.

## Figures and Tables

**Table 1:** Pairwise comparisons between groups using Sidak’s correction for the percentage of body weight variation based on the estimated marginal means for the percentage of body weight variation post-surgery.

Comparison between Groups	Mean Difference	SEM	*p*-Value
Sham aged rats vs. 6-OHDA aged rats	7.88	2.40	*p* = 0.019
Sham aged rats vs. sham young adult rats	−15.1	2.61	*p* < 0.0001
Sham aged rats vs. 6-OHDA young adult rats	−4.43	2.46	*p* = 0.410
6-OHDA aged rats vs. 6-OHDA young adult rats	−12.3	2.09	*p* < 0.0001
Sham young adult rats vs. 6-OHDA young adult rats	10.7	2.33	*p* = 0.001
Sham young adult rats vs. 6-OHDA aged rats	23.0	2.27	*p* < 0.0001

**Table 2:** Pairwise comparisons between groups using Sidak’s correction for the cylinder test based on the estimated marginal means for the percentage of touches using the contralateral forelimb.

Comparison between Groups	Mean Difference	SEM	*p*-Value
Sham aged rats vs. 6-OHDA aged rats	71.3	4.78	*p* < 0.0001
Sham aged rats vs. Sham young adult rats	−3.39	4.99	0.986
Sham aged rats vs. 6-OHDA young adult rats	67.3	4.99	*p* < 0.0001
6-OHDA aged rats vs. 6-OHDA young adult rats	−4.05	4.78	0.958
Sham young adult rats vs. 6-OHDA young adult rats	70.7	4.99	*p* < 0.0001
Sham young adult rats vs. 6-OHDA aged rats	74.7	4.78	*p* < 0.0001

**Table 3:** Pairwise comparisons between groups using Sidak’s correction for the staircase test based on the estimated marginal means for the percentage of the success rate using the contralateral forelimb.

**Staircase**			
**Comparison between Groups**	**Mean Difference**	**SEM**	***p*-Value**
Sham aged rats vs. 6-OHDA aged rats	57.9	6.15	*p* < 0.0001
Sham aged rats vs. sham young adult rats	0.127	6.35	*p* = 1.0
Sham aged rats vs. 6-OHDA young adult rats	39.7	6.15	*p* < 0.0001
6-OHDA aged rats vs. 6-OHDA young adult rats	−18.3	5.02	*p* = 0.013
Sham young adult rats vs. 6-OHDA young adult rats	36.6	5.26	*p* < 0.0001
Sham young adult rats vs. 6-OHDA aged rats	57.8	5.26	*p* < 0.0001
**Staircase–Forced-Choice**			
**Comparison between Groups**	**Mean Difference**	**SEM**	***p*-Value**
Sham aged rats vs. 6-OHDA aged rats	58.7	6.22	*p* < 0.0001
Sham aged rats vs. sham young adult rats	0.061	6.43	*p* = 1.0
Sham aged rats vs. 6-OHDA young adult rats	41.1	6.22	*p* < 0.0001
6-OHDA aged rats vs. 6-OHDA young adult rats	−17.6	5.08	*p* = 0.019
Sham young adult rats vs. 6-OHDA young adult rats	41.1	5.33	*p* < 0.0001
Sham young adult rats vs. 6-OHDA aged rats	58.7	5.33	*p* < 0.0001

## Abbreviations

PD	Parkinson’s disease
6-OHDA	6-hydroxydopamine
SNpc	Substantia nigra pars compacta
DA neurons	Dopamine-containing neurons
MFB	Medial forebrain bundle
SEM	Standard error of the mean
TH	Tyrosine hydroxylase
STR	Striatum
NADPH	Nicotinamide adenine dinucleotide phosphate
SN	Substantia nigra
MPTP	1-methyl-4-phenyl-1,2,3,6-tetrahydropyridine
BDNF	Brain-derived neurotrophic factor
GDNF	Glial cell line-derived neurotrophic factor
PFA	Paraformaldehyde
PBS	Phosphate-buffered saline
FCS	Fetal calf serum
RT	Room temperature
PBS-T	Phosphate-buffered saline -Triton
DAB	3,3’- diaminobenzidine tetrahydrochloride

## References

[1] Pringsheim T., Jette N., Frolkis A., Steeves T.D. (2014). The prevalence of Parkinson’s disease: A systematic review and meta-analysis. Mov. Disord..

[2] Poewe W., Seppi K., Tanner C.M., Halliday G.M., Brundin P., Volkmann J. (2017). Parkinson disease. Nat. Rev. Dis. Primers.

[3] Klingelhoefer L., Reichmann H. (2015). Pathogenesis of Parkinson disease—The gut–brain axis and environmental factors. Nat. Rev. Neurol..

[4] Spatola M., Wider C. (2014). Genetics of Parkinson’s disease: The yield. Parkinsonism Relat. Disord..

[5] Pang S.Y.-Y., Ho P.W.-L., Liu H.-F., Leung C.-T., Li L., Chang E.E.S., Ramsden D.B., Ho S.-L. (2019). The interplay of aging, genetics and environmental factors in the pathogenesis of Parkinson’s disease. Transl. Neurodegener..

[6] Collier T.J., Kanaan N.M., Kordower J.H. (2011). Ageing as a primary risk factor for Parkinson’s disease: Evidence from studies of non-human primates. Nat. Rev. Neurosci..

[7] Reeve A., Simcox E., Turnbull D. (2014). Ageing and Parkinson’s disease: Why is advancing age the biggest risk factor?. Ageing Res. Rev..

[8] Lotti M. (2002). Age-related sensitivity of the nervous system to neurotoxic insults. Toxicol. Lett..

[9] Phinney A.L., Andringa G., Bol J.G., Wolters E.C., van Muiswinkel F.L., van Dam A.-M.W., Drukarch B. (2006). Enhanced sensitivity of dopaminergic neurons to rotenone-induced toxicity with aging. Parkinsonism Relat. Disord..

[10] Boger H.A., Middaugh L.D., Zaman V., Hoffer B., Granholm A.-C. (2008). Differential effects of the dopamine neurotoxin MPTP in animals with a partial deletion of the GDNF receptor, GFRα1, gene. Brain Res..

[11] Jankovic J., Aguilar L.G. (2008). Current approaches to the treatment of Parkinson’s disease. Neuropsychiatr. Dis. Treat..

[12] Oertel W., Schulz J.B. (2016). Current and experimental treatments of Parkinson disease: A guide for neuroscientists. J. Neurochem..

[13] Teixeira F.G., Carvalho M.M., Panchalingam K.M., Rodrigues A.J., Mendes-Pinheiro B., Anjo S., Manadas B., Behie L.A., Sousa N., Salgado A.J. (2017). Impact of the secretome of human mesenchymal stem cells on brain structure and animal behavior in a rat model of Parkinson’s disease. Stem Cells Transl. Med..

[14] Mendes-Pinheiro B., Teixeira F.G., Anjo S.I., Manadas B., Behie L.A., Salgado A.J. (2018). Secretome of undifferentiated neural progenitor cells induces histological and motor improvements in a rat model of Parkinson’s disease. Stem Cells Transl. Med..

[15] Pinheiro B.M., Anjo S.I., Manadas B., da Silva J.D., Marore A., Teixeira F.G., Salgado A.J. (2019). Bone marrow mesenchymal stem cells’ secretome exerts neuroprotective effects in a Parkinson’s disease rat model. Front. Bioeng. Biotechnol..

[16] De Jesús-Cortés H., Miller A.D., Britt J.K., DeMarco A.J., De Jesús-Cortés M., Stuebing E., Naidoo J., Vázquez-Rosa E., Morlock L., Williams N.S. (2015). Protective efficacy of P7C3-S243 in the 6-hydroxydopamine model of Parkinson’s disease. NPJ Parkinson’s Dis..

[17] Lang A.E., Espay A.J. (2018). Disease modification in Parkinson’s disease: Current approaches, challenges, and future considerations. Mov. Disord..

[18] Dawson T.M., Golde T.E., Lagier-Tourenne C. (2018). Animal models of neurodegenerative diseases. Nat. Neurosci..

[19] Duty S., Jenner P. (2011). Animal models of Parkinson’s disease: A source of novel treatments and clues to the cause of the disease. Br. J. Pharmacol..

[20] Ricaurte G., DeLanney L., Finnegan K., Irwin I., Langston J. (1988). The dopamine-depleting effect of 6-hydroxydopamine does not increase with aging. Brain Res..

[21] Cass W.A., Harned M.E., Bailey S.L. (2002). Enhanced effects of 6-hydroxydopamine on evoked overflow of striatal dopamine in aged rats. Brain Res..

[22] Cass W.A., Peters L.E., Smith M.P. (2005). Reductions in spontaneous locomotor activity in aged male, but not female, rats in a model of early Parkinson’s disease. Brain Res..

[23] Tamás A., Lubics A., Szalontay L., Lengvári I., Reglődi D. (2005). Age and gender differences in behavioral and morphological outcome after 6-hydroxydopamine-induced lesion of the substantia nigra in rats. Behav. Brain Res..

[24] Tamás A., Lubics A., Lengvári I., Reglődi D. (2006). Effects of age, gender, and gonadectomy on neurochemistry and behavior in animal models of Parkinson’s disease. Endocrine.

[25] Villar-Cheda B., Valenzuela R., Rodriguez-Perez A.I., Guerra M.J., Labandeira-Garcia J.L. (2012). Aging-related changes in the nigral angiotensin system enhances proinflammatory and pro-oxidative markers and 6-OHDA-induced dopaminergic degeneration. Neurobiol. Aging.

[26] Carvalho M.M., Campos F.L., Coimbra B., Pêgo J.M., Rodrigues C., Lima R., Rodrigues A.J., Sousa N., Salgado A.J. (2013). Behavioral characterization of the 6-hydroxidopamine model of Parkinson’s disease and pharmacological rescuing of non-motor deficits. Mol. Neurodegener..

[27] Ma Y., Zhan M., OuYang L., Li Y., Chen S., Wu J., Chen J., Luo C., Lei W. (2014). The effects of unilateral 6-OHDA lesion in medial forebrain bundle on the motor, cognitive dysfunctions and vulnerability of different striatal interneuron types in rats. Behav. Brain Res..

[28] Teixeira F.G., Vilaça-Faria H., Domingues A.V., Campos J., Salgado A.J. (2020). Preclinical Comparison of Stem Cells Secretome and Levodopa Application in a 6-Hydroxydopamine Rat Model of Parkinson’s Disease. Cells.

[29] Rentsch P., Stayte S., Morris G.P., Vissel B. (2019). Time dependent degeneration of the nigrostriatal tract in mice with 6-OHDA lesioned medial forebrain bundle and the effect of activin A on l-Dopa induced dyskinesia. BMC Neurosci..

[30] Seidler R.D., Bernard J.A., Burutolu T.B., Fling B.W., Gordon M.T., Gwin J.T., Kwak Y., Lipps D.B. (2010). Motor control and aging: Links to age-related brain structural, functional, and biochemical effects. Neurosci. Biobehav. Rev..

[31] Hoogendam Y.Y., van der Lijn F., Vernooij M.W., Hofman A., Niessen W.J., van der Lugt A., Ikram M.A., van der Geest J.N. (2014). Older age relates to worsening of fine motor skills: A population-based study of middle-aged and elderly persons. Front. Aging Neurosci..

[32] Voelcker-Rehage C. (2008). Motor-skill learning in older adults—A review of studies on age-related differences. Eur. Rev. Aging Phys. Act..

[33] Barnéoud P., Descombris E., Aubin N., Abrous D.N. (2000). Evaluation of simple and complex sensorimotor behaviours in rats with a partial lesion of the dopaminergic nigrostriatal system. Eur. J. Neurosci..

[34] Zucca F.A., Segura-Aguilar J., Ferrari E., Muñoz P., Paris I., Sulzer D., Sarna T., Casella L., Zecca L. (2017). Interactions of iron, dopamine and neuromelanin pathways in brain aging and Parkinson’s disease. Prog. Neurobiol..

[35] Collier T.J., Kanaan N.M., Kordower J.H. (2017). Aging and Parkinson’s disease: Different sides of the same coin?. Mov. Disord..

[36] Surmeier D.J. (2018). Determinants of dopaminergic neuron loss in Parkinson’s disease. FEBS J..

[37] Sugama S., Yang L., Cho B.P., DeGiorgio L.A., Lorenzl S., Albers D.S., Beal M.F., Volpe B.T., Joh T.H. (2003). Age-related microglial activation in 1-methyl-4-phenyl-1, 2, 3, 6-tetrahydropyridine (MPTP)-induced dopaminergic neurodegeneration in C57BL/6 mice. Brain Res..

[38] Ali S., David S., Newport G. (1993). Age-related susceptibility to MPTP-induced neurotoxicity in mice. Neurotoxicology.

[39] Collier T.J., Lipton J., Daley B.F., Palfi S., Chu Y., Sortwell C., Bakay R.A., Sladek J.R., Kordower J.H. (2007). Aging-related changes in the nigrostriatal dopamine system and the response to MPTP in nonhuman primates: Diminished compensatory mechanisms as a prelude to parkinsonism. Neurobiol. Dis..

[40] Wang X., Guan Q., Wang M., Yang L., Bai J., Yan Z., Zhang Y., Liu Z. (2015). Aging-related rotenone-induced neurochemical and behavioral deficits: Role of SIRT2 and redox imbalance, and neuroprotection by AK-7. Drug Des. Dev. Ther..

[41] Ureshino R.P., Costa A.J., Erustes A.G., Pereira G.J.D.S., Sinigaglia-Coimbra R., Smaili S.S. (2018). Effects of aging in the striatum and substantia nigra of a Parkinson’s disease animal model. Toxicol. Pathol..

[42] Yurek D.M., Fletcher-Turner A. (2000). Lesion-induced increase of BDNF is greater in the striatum of young versus old rat brain. Exp. Neurol..

[43] Yurek D.M., Fletcher-Turner A. (2001). Differential expression of GDNF, BDNF, and NT-3 in the aging nigrostriatal system following a neurotoxic lesion. Brain Res..

[44] Collier T.J., Ling Z.D., Carvey P.M., Fletcher-Turner A., Yurek D.M., Sladek J.R., Kordower J.H. (2005). Striatal trophic factor activity in aging monkeys with unilateral MPTP-induced parkinsonism. Exp. Neurol..

[45] Choi D.-Y., Zhang J., Bing G. (2010). Aging enhances the neuroinflammatory response and α-synuclein nitration in rats. Neurobiol. Aging.

[46] Gordon M.N., Schreier W.A., Ou X., Holcomb L.A., Morgan D.G. (1997). Exaggerated astrocyte reactivity after nigrostriatal deafferentation in the aged rat. J. Comp. Neurol..

[47] Koprich J.B., Reske-Nielsen C., Mithal P., Isacson O. (2008). Neuroinflammation mediated by IL-1β increases susceptibility of dopamine neurons to degeneration in an animal model of Parkinson’s disease. J. Neuroinflamm..

[48] Singh S., Ahmad R., Mathur D., Sagar R.K., Krishana B., Arora R., Sharma R.K. (2006). Neuroprotective effect of BDNF in young and aged 6-OHDA treated rat model of Parkinson disease. Indian J. Exp. Biol..

[49] Grimmig B., Daly L., Subbarayan M., Hudson C., Williamson R., Nash K., Bickford P.C. (2018). Astaxanthin is neuroprotective in an aged mouse model of Parkinson’s disease. Oncotarget.

[50] Sortwell C.E., Camargo M.D., Pitzer M.R., Gyawali S., Collier T.J. (2001). Diminished survival of mesencephalic dopamine neurons grafted into aged hosts occurs during the immediate postgrafting interval. Exp. Neurol..

[51] Misal U.S., Joshi S.A., Shaikh M.M. (2016). Delayed recovery from anesthesia: A postgraduate educational review. Anesth. Essays Res..

[52] Chemali J., Kenny J., Olutola O., Taylor N., Kimchi E., Purdon P., Brown E., Solt K. (2015). Ageing delays emergence from general anaesthesia in rats by increasing anaesthetic sensitivity in the brain. Br. J. Anaesth..

[53] Paxinos G., Watson C. (2009). The Rat Brain in Stereotaxic Coordinates: Compact.

[54] Schallert T., Tillerson J.L. (2000). Intervention strategies for degeneration of dopamine neurons in parkinsonism. Central Nervous System Diseases.

[55] Heuer A., Smith G.A., Lelos M.J., Lane E.L., Dunnett S.B. (2012). Unilateral nigrostriatal 6-hydroxydopamine lesions in mice I: Motor impairments identify extent of dopamine depletion at three different lesion sites. Behav. Brain Res..

[56] Montoya C., Campbell-Hope L., Pemberton K., Dunnett S. (1991). The “staircase test”: A measure of independent forelimb reaching and grasping abilities in rats. J. Neurosci. Methods.

[57] Campos F.L., Carvalho M.M., Cristovão A.C., Je G., Baltazar G., Salgado A.J., Kim Y.-S., Sousa N. (2013). Rodent models of Parkinson’s disease: Beyond the motor symptomatology. Front. Behav. Neurosci..

